# Live Music Performance: The Relationship Between Flow and Music Performance Anxiety

**DOI:** 10.3389/fpsyg.2021.725569

**Published:** 2021-11-25

**Authors:** Claudia Spahn, Franziska Krampe, Manfred Nusseck

**Affiliations:** ^1^Freiburg Institute for Musicians’ Medicine, University Medical Center Freiburg, University of Music Freiburg, Medical Faculty of the Albert-Ludwigs-University Freiburg, Freiburg Center for Research and Teaching in Music, Freiburg, Germany; ^2^Children’s Hospitals Schwabing and Harlaching Munich, Hospital Rechts der Isar, Technical University Munich, Munich, Germany

**Keywords:** performance science, music performance anxiety, flow, professional musicians, amateur musicians

## Abstract

Most studies exploring the relation between flow and Music Performance Anxiety (MPA) have focused on the disposition of generally experiencing flow and the occurrence of MPA. Little is known about the connection between experiencing flow and MPA as it relates to a specific performance. In this study, flow and MPA have been investigated in 363 orchestral musicians in relation to a particular live music performance. The musicians were asked to fill out a questionnaire immediately after a concert. Flow experience during the performance was measured using the Flow Short Scale. The Performance-specific Questionnaire on MPA (PQM) was used for MPA. The PQM addresses particular aspects of MPA and refers retrospectively to the time before and during the performance as well as to the moment of filling out the questionnaire after the performance. Using three scales, the functional coping, the perceived symptoms of MPA and self-efficacy were determined for each time point of the performance. The results showed that experiencing flow was on average higher among orchestral musicians compared to a sample of the general population. However, there were differences between the professional and non-professional musicians. All PQM scales showed significant correlations with the global flow scale. Regression analysis on the global flow score found that regarding the time before the performance the PQM scale symptoms of MPA were diametrically connected with the flow experience. The PQM scale functional coping was shown to be positively related to the flow during the performance. Moreover, high self-efficacy was found to be closely related with stronger flow experience. Furthermore, flow seems to have positive effects on functionally coping with MPA and the self-efficacy after the performance. These findings confirm the negative relationship between flow and symptoms of MPA, offering further approaches in understanding the relationship especially for live music performances.

## Introduction

### Flow and Musical Performance

The concept of flow was introduced by Csikszentmihalyi (1975; 1990; 1997) to describe a specific experience, whereby a participant is completely involved in the activity and unaware of their surroundings. Within this moment, increased focus and intense concentration can be experienced, with a certain awareness of one’s merging with the action (Abuhamdeh, 2020). This state is characterized by a sense of complete absorption in the present activity with an effortless and automatic execution of the action being performed, including an altered experience of time (Nakamura and Csikszentmihalyi, 2002). As the experience of flow is commonly associated with a certain degree of facility and high quality in carrying out the action concerned, it is often linked to peak performances and referred to as an optimal psychophysical state (Biasutti, 2017). Flow creates a feeling of satisfaction and positive self-confidence (Moneta, 2004; Rheinberg et al., 2007). It is also positively related to subjective well-being (Smolej-Fritz and Avsec, 2007), even in times of the coronavirus pandemic (Habe et al., 2021).

Flow experiences occur frequently while making music. Musical activities are acknowledged to contain intense flow experiences (Lowis, 2002). Musicians often report beneficial effects on the performance as a result of experiencing flow. Bloom and Skutnick-Henley (2005) identified particular features regularly experienced by musicians during flow. The most important characteristics were self-confidence and self-belief while playing, as well as the desire to experience and express feelings through music. It has also been found that more flow was experienced when playing in an orchestra or singing in a chorus than in performing solo (Smolej-Fritz and Avsec, 2007). Musicians showed stronger flow experiences when playing in the presence of an audience compared to without an audience (Deutsch et al., 2009).

Regarding the level of musical expertise, flow was not found to be experienced more often among trained elite musicians, i.e., those who have studied music, compared to amateur musicians (Sinnamon et al., 2012). However, a considerable skill in the activity is required for flow to be experienced (Rheinberg, 2010). Therefore, a certain degree of playing experience is necessary to encounter flow (Marin and Bhattacharya, 2013). Cohen and Bodner (2021) examined the individual’s disposition to experience flow with the Dispositional Flow Scale–2 in professional musicians. The questionnaire enables a description of how often the respondents experience different dimensions of flow in their musical activity. The seven-stage response scale was converted into a dichotomous statement of high and low frequency of flow. Based on this evaluation, the authors concluded that 85.5% of the 202 professional orchestral musicians reported high frequencies of flow. Additionally, they found that these dispositional flow findings of professional orchestral musicians were higher compared to music students.

In a qualitative study, Antonini Philippe et al. (2021) interviewed high-level musicians to get their perspectives on what contributes to the occurrence of flow during a music performance. They found several factors that promote the emergence of flow, such as social support, performance preparation, connection with one’s body, awareness of skills and self-confidence, intrinsic motivation, attentional focus, and transcendence. In particular, the feeling of competence and self-confidence was an important factor. Confidence in one’s own abilities is a predictor of successful performances (Papageorgi et al., 2007) and musicians with greater self-confidence are more likely to be able to focus fully on the performance and may facilitate flow (Csikszentmihalyi, 1990). Another important factor that supports flow is motivation (Antonini Philippe et al., 2021), the desire to play and to perform with full commitment.

When examining the experience of flow during the live performance of music before an audience, one has to consider also the performance-specific and individual degrees of Music Performance Anxiety (MPA) integral to such situations. Wrigley and Emmerson (2013) measured the flow as experienced by music students immediately after their live examination performances. They found the flow to be rather low and suggest that performance anxiety in this highly demanding situation may have negatively interfered with the subjective flow attainable. In addition, they found no evidence of an association between flow with either instrument or gender. This, however, may possibly be caused by the specific situation of the performance. Other studies confirm that there is evidence of no effect of gender on dispositional flow among students and professionals (Marin and Bhattacharya, 2013; Cohen and Bodner, 2019). In contrast, dispositional flow was found to be experienced more in male music students than in female students (Habe et al., 2019, 2021). They also found that the dispositional flow was higher for group than for individual performances. However, so far, it has not been investigated whether differences exist in the flow experiences between different instruments and gender during typical live performances of ensembles and orchestras.

### Music Performance Anxiety

MPA is defined as a particular psychophysical state that is connected to certain forms of anxiety (Kenny, 2011; Spahn, 2015). Typically, MPA is accompanied by increased body tension, concentration and attention, and by intensified emotional experiences. It can be assumed that everyone can experience MPA and that it is part of the human repertoire of experiences and should not be considered pathological *per se*.

However, depending on its severity, MPA has both facilitating and debilitating potential in terms of performance outcomes (Osborne and Franklin, 2002). At a supportive level, MPA acts as performance enhancing and can raise the performance quality. In certain cases, it can even support some kind of performance boost (Simoens et al., 2015; González et al., 2018). On a higher level, MPA can impair the performance due to overwhelming aspects of anxiety (Spahn, 2015). Debilitating MPA accompanied by perceived pressure have been found to impact negatively on musical performance success (Simoens et al., 2015). Therefore, it is important for musicians to develop skills to successfully cope with MPA and to regulate physiological arousal and the potential debilitating effects of MPA (Papageorgi et al., 2007). It has been shown that negative coping strategies such as the need for social approval and avoidance and denial have positive correlations with MPA in advanced and professional musicians (Biasutti and Concina, 2014).

MPA is influenced in its occurrence by a variety of factors. As a general factor, the anxiety found in the individual as an innate personality trait contributes to the development of performance-impairing MPA (Kenny, 2011). However, individual performance experiences play a crucial role, too, and can add to the negative effects of MPA.

Other influencing factors arise from the context of the performance. It has been shown that in concerts MPA increased with the size of the audience, the performance environment, and the personal importance attached to the performance (Le Blanc et al., 1997). The difficulty of the performance is also associated with MPA (Kenny, 2011). The more difficult the performance was perceived, the higher the MPA. In contrast, the musical self-efficacy, i.e., the belief in one’s own capabilities to make a success of the performance, can act as a supportive factor in dealing with MPA (McCormick and McPherson, 2003; McPherson and McCormick, 2006; Spahn et al., 2021). Furthermore, MPA is rather independent of musical training and expertise, as musically experienced and well-prepared players also showed high degrees of MPA that were more related to the importance of the performance (Yoshie et al., 2009).

Within a musical performance context, MPA needs to be considered as a process over time, especially before, during and after the performance (Papageorgi et al., 2007; Spahn et al., 2021). Each of these performance times provides different aspects concerning the occurrence and development of MPA. Before the performance, final preparations for the performance are being made and the anticipation rises. At that time, MPA is often described as highest, with its influence at its greatest. Kalenìska-Rodzaj (2018) showed that the personal satisfaction of musicians and their assessment of the musical quality was significantly better when they had an average MPA rather than a high MPA prior to the performance.

The time after the performance is also a very important moment. While the feelings before an upcoming performance can influence the performance itself, the impression and judgment of a just given performance can affect future performances, too. A negative perception after a performance can cause low self-esteem and low self-efficacy, which may result in increased MPA in subsequent performances. Conversely, perceiving the performance as positive can enhance confidence and self-efficacy, creating an optimistic foundation for performances in the future (Papageorgi et al., 2007).

### Flow and Music Performance Anxiety

Since the experience of flow and MPA occur together in musical performances, the question arises as to how both flow and MPA are related to each other. As one could expect, it has been found that the degree of MPA and the tendency to experience flow have a significant negative correlation (Kirchner et al., 2008). The authors declared that a higher degree of MPA reduces the possibility of experiencing flow. They also claimed that a particular influencing factor could be self-confidence, as it reduces MPA and increases the occurrence of flow.

Fullagar et al. (2013) showed that flow and MPA are incompatible states, in which the occurrence of one reduces that of the other. In a more recent study, the relationship between flow and MPA in professional classical orchestral musicians was investigated by using a hierarchical regression analysis (Cohen and Bodner, 2021). The results confirmed the negative connection between flow and MPA and the authors suggested that facilitating flow might provide the possibility to reduce MPA. Since MPA is inversely correlated with self-efficacy, it might be possible that self-efficacy is also associated with the occurrence of flow. Feeling confident in one’s own abilities can have a positive effect on flow experiences.

Kutepova-Bredun (2018) investigated probable differences in the experience of flow of elite and amateur musicians. Their study was represented by 60 musicians in Dnipro (Ukraine) aged 18–60, with half of them being students of the musical college, students of the conservatory and representatives of the teaching staff of the musical school and the other half being amateur musicians (other students of the University). She found no statistically meaningful differences between both groups in the general level of the flow state; however, there was a statistically higher value in the fluency scale among the elite musicians compared to the amateur musicians. The author interprets her results to mean that musical activity is perceived more fluently by advanced musicians because of their higher levels of musical skills and abilities.

In general, anxiety can be experienced when the challenge of the task is perceived to be high and one’s own skills compared to the task are perceived as low (Csikszentmihalyi, 1975). For MPA, the difficulty follows a linear relation, where low MPA can be found when the difficulty is low and high MPA when the performance is more demanding (Kenny, 2011). In contrast, a requirement for experiencing flow is the ideal balance between challenge and skill (Engeser and Rheinberg, 2008). In low challenging tasks, the flow experiences are found rather rarely (Rheinberg and Vollmeyer, 2003). In such cases, the task may be perceived as dull as it does not need much skill, and may lead to the appearance of boredom (Csikszentmihalyi, 1975). However, the feeling of fluency in such performances can be high. If the task is too difficult compared to one’s own skill, flow also occurs rather seldom as the focus is high on fulfilling the task. Most flow experiences have been found where a good balance between demands and skills is present. These findings showed that flow and difficulty build an inverted U-shape relation (Rheinberg et al., 2003).

Studies investigating the relation between MPA and flow have either measured the dispositional flow or evaluated flow at a particular performance and correlated the flow values with the general disposition of experiencing MPA. As flow exists only when the activity is performed, the measuring of flow should be addressed to that particular situation. In musical performances, the progression and degree of MPA plays an important role. Hence, it would be interesting to investigate flow and MPA with regard to a specific live performance. In this context, the perception of MPA during the period prior to the performance could be important in predicting the occurrence of flow as the setting for the performance arises. During the performance, both flow and MPA may exist simultaneously and can interact with each other. The experiences of MPA in a performance reported after said performance could also be related to flow in an opposite way, such that the flow experience may influence the impressions of the performance.

Therefore, in this study the performance-specific questionnaire on MPA (PQM; Spahn et al., 2016) was used and linked to the flow experience (Flow Short-Scale; Rheinberg et al., 2003) of a particular performance. The PQM questionnaire addresses certain aspects of MPA such as the functional coping, the occurrence of symptoms of MPA and the self-efficacy, and has to be filled in immediately after a given performance. The questionnaire considers retrospectively the time before and during the performance and the time of completion of the questionnaire.

With this design, flow during a real performance and specific relations with the flow experience can be investigated concerning symptoms of MPA, functional coping and self-efficacy before, during and after the performance.

All together the following questions are to be examined in the present study:

1. To what extent do orchestral musicians experience flow during a live performance?
2. Are there differences in the occurrence of flow between professional and non-professional instrumentalists playing in an orchestra?
3. Are there differences of flow between different instrumentalists?
4. How does the experience of flow during a live performance correlate with aspects of MPA (i.e., functional coping, symptoms of MPA, and self-efficacy) before, during and after this performance?
5. Can the perception of MPA before and during the performance predict the occurrence of flow?

In this study, musicians who performed in a live concert in front of a public audience have been investigated in terms of flow and MPA experiences. Regarding the questions, it was hypothesized that the MPA during the performance would show the already known negative relation with the flow experience. It was also assumed that a higher level of MPA before the performance might have a negative effect on flow occurrences during the performance. After the performance, the existence of flow during the performance could possibly have a positive effect on the self-efficacy and the player’s judgment of the quality of the music.

## Materials and Methods

### Participants

The sample taken for this study consists of 363 classical orchestral musicians. The orchestras included were either professional orchestras (e.g., radio symphony orchestras) containing musicians with a professional musical background (i.e., university or conservatory music degree) or non-professional orchestras with semi-professional musicians without any music university education who play as a hobby. All the orchestras were known locally, with fixed lineups playing standard repertoire concerts. Depending on the specific information pertaining to each orchestra, the musicians were divided, respectively, into professional and non-professional musicians (Table 1).

**TABLE 1:** Sample description with statistical differences.

	Professional musicians (*n* = 136)	Non-professional musicians (*n* = 227)	Total sample (*n* = 363)	Difference between the groups
Percentage of the total sample	38%	62%	100%	
Age in years mean (SD)	43.5 (12.9)	26.5 (10.9)	32.8 (14.3)	*p* < 0.001
Gender (female)	40.6%	56.2%	50.4%	*p* = 0.004
Instruments				*p* = 0.021
Strings (*n* = 215)	75%	60%	65%	
Woodwinds (*n* = 58)	13%	20%	18%	
Brass (*n* = 43)	7%	16%	13%	
Percussion (*n* = 15)	5%	4%	4%	

Thus, the study included 38% professional and 62% non-professional orchestra musicians. The mean age was 32.8 years (*SD* = 14.3 years) and the professional musicians were significantly older than the non-professional musicians [*F*_(1, 361)_ = 173.30, *p* < 0.001]. In the total sample, 50.4% were female musicians. The gender distribution was significantly different between the professional and non-professional musicians with more female musicians in the non-professional group [χ^2^_(359, 1)_ = 8.14, *p* = 0.004]. The instrumentalists were divided into different groups of instruments. Particular instruments that rarely occurred (8%) were excluded. The total instrumental distribution was 65% strings, 18% woodwinds, 13% brass, and 4% percussion. There was a significant distribution difference of instruments between the professional and non-professional musicians [χ^2^_(331, 3)_ = 9.76, *p* = 0.021].

### Measures

A questionnaire was constructed that included general questions about gender, age, and the main instrument, as well as standardized self-assessment questionnaires regarding MPA and flow, which are described below.

#### Flow Experience

Flow has been measured by using the Flow Short-Scale (Rheinberg et al., 2003). It was considered as a self-perceived experience on a continuous scale assessing retrospectively the feeling of having experienced flow in a just performed task. It is therefore a measure of situational flow and refers in this study to the past musical performance. The questionnaire consists of 10 items addressing certain flow statements which had to be rated on a seven-point scale (1—“I don’t agree” to 7—“I totally agree”). All items were used to calculate the Global Flow Score, with higher values representing a higher level of an overall flow experience (Cronbach’s α = 0.82). A mean value of the Global Flow Score in a large general population (*n* = 4,479) across various different activities was at *M* = 4.97 (Rheinberg et al., 2005).

In addition, the questionnaire contains two subscales (Engeser and Rheinberg, 2008). Six items describe the fluency of the performance, referring to a perceived automated processing of the activity (e.g., “My performance ran fluidly and smoothly”). Higher values indicate a more fluent performance (Cronbach’s α = 0.86) and the mean value in the general population was at *M* = 5.4 (Rheinberg et al., 2005).

The second subscale addresses the absorption in the activity. It also includes the feeling of not noticing how time elapses (e.g., “I didn’t notice time passing”). Higher values describe a more intensive absorption in the activity (Cronbach’s α = 0.64) and the mean value in the general population was found at *M* = 4.3 (Rheinberg et al., 2005).

#### Self-Reported Music Performance Anxiety

MPA was measured using the German self-assessment questionnaire “Fragebogen zum Auftritt für MusikerInnen” (FZAM; Spahn et al., 2016), the so-called PQM (Performance-specific Questionnaire on Music Performance Anxiety). The questionnaire contains a total of 42 items that address different dimensions of MPA-related areas. Filling out the questionnaire is done immediately after a musical performance. The participants are asked to answer retrospectively questions about the time directly before the performance and during the performance, as well as questions regarding the time spent filling out the questionnaire after the performance. The first 32 items are composed of three scales, which are also divided into the different performance times: (1) functional coping (Cronbach’s α = 0.74), considering positive activities in handling MPA (e.g., “I managed to control my agitation and stay calm”), (2) symptoms of MPA (Cronbach’s α = 0.77), describing the occurrence of MPA-specific implications (e.g., “I could sense signs of agitation in my body”), and (3) self-efficacy (Cronbach’s α = 0.73), addressing one’s own confidence in performing (e.g., “I was looking forward to going on stage and showing what I could do”). The answers were provided on a five-point Likert scale and the scale values were calculated as the mean of the items. Higher scores in the functional coping and self-efficacy scales indicate better coping and higher self-efficacy. Higher scores in the symptoms of MPA scale indicate higher levels of debilitating MPA. The reliability of the preperformance scales had been validated by using a state anxiety questionnaire that was filled out directly before the performance and showed significant correlations with the variables referring to the time before the performance (Biwer, 2015).

The following seven questions concerned a self-assessment of the musical quality of the performance. The items addressed several music-related aspects such as intonation, dynamics, and expression and were answered on a six-point scale ranging from 1—“very poor” to 6—“excellent.” The mean of the items was calculated as a scale of the musical quality (Cronbach’s α = 0.88).

In the last three questions, the musicians were asked to rate the relative importance and difficulty of the performance. In the first question, the participants had to indicate the importance they attached to the performance on a four-point scale (“Doing well in this concert was personally…” with 1—“…not important to me” to 4—“…very important to me”). The second question used a four-point scale to ask about the performance difficulty compared to other performances (“Compared to other performances this performance was…” with 1—“…easy for me” to 4—“…difficult for me”). In the third question, the musicians had to specify the individual difficulty of the concert on a five-point answer scale (“The difficulty of this concert was…” with 1—“too low,” 3—“just right” to 5—“too high”). The difference between the last two questions was in order to rate the difficulty using different reference points. The compared difficulty asked respondents to judge the general difficulty with reference to the concert program and the orchestral situation, as well as the individual difficulty taking into account personal demands made on the player.

### Procedure

The musicians were asked to fill out the questionnaire of this study directly after a performed concert. The concerts were held in public with audiences ranging between 300 and 1,500 persons and provided a regular program according to the standard performance repertoire of the orchestra. The questionnaire was given to the musicians before they entered the off-stage facilities. They were asked to fill out the questionnaire before talking with other people or musicians about the concert. After they completed it, the questionnaire was collected by the experimenters. The questionnaire was completely anonymous, and participation was voluntary. There was no payment in return for participation. The orchestras were informed about the study and the participation procedure in the dress rehearsal prior to the concert. The study was ethically approved by the Ethics Committee of the University Medical Center Freiburg.

### Data Analyses

The data analyses were performed using SPSS (Version 26, Armonk, NY: IBM Corp.). For each variable, descriptive statistics were calculated. Distribution differences of non-parametric variables were performed with cross tables and Pearson’s Chi-square was reported. The scales of the PQM and flow showed insignificant Kolmogorov-Smirnov tests indicating normal distributions in all scales. Parametric comparisons of the PQM and the flow scales have been calculated with multivariate ANOVAs. On significance, *post hoc* analyses with Tukey HSD correction were performed. Comparisons of scale values with mean values of the general population were carried out using simple *t*-Tests. Correlations were reported with the Pearson’s r coefficient and the level of significance. Correlations between ordinal-scaled variables were using the Kendall Tau correlation. To investigate the relationship between particular parameters before and during the performance and the flow experience, linear hierarchical regression analyses with enter inclusion method were used. The adjusted *R*^2^ was calculated as a value for the explained variance. For all variables, the standardized beta coefficient and the *p*-values were reported. Effect sizes were reported by using Cohen’s *d* (Cohen, 1988). The level of statistical significance was set at 0.05.

## Results

### Experience of Flow

The mean values of the flow scales are shown in Table 2. The mean Global Flow Score across all musicians was 5.09 (*SD* = 0.93). Addressing the second research question, there was no significant difference in this scale between professional and non-professional musicians. Considering the extent of the flow experience, the flow scale values were compared with the mean values of the general population (Rheinberg et al., 2005). The mean value of the Global Flow Scale in the total sample was significantly higher compared to the mean value of the general population [*t*(362) = 2.47, *p* = 0.014, *d* = 0.26]. However, the value of the professional musicians was not significantly different to the value in the general population [*t*(135) = 0.93, *n.s.*]; only the mean value of the Global Flow Score among the non-professional musicians was significantly higher compared to the value in the general population [*t*(226) = 2.46, *p* = 0.015, *d* = 0.33].

**TABLE 2:** Mean values of the Flow Short-Scale (Rheinberg et al., 2003) by total sample and subgroups with statistical differences (n.s., not significant; in bold: highest values).

	Professional musicians (*n* = 136) mean (SD)	Non-professional musicians (*n* = 227) mean (SD)	Total sample (*n* = 363) mean (SD)	Statistical difference between subgroups
Global Flow Score	5.05 (0.99)	5.11 (0.89)	5.09 (0.93)	n.s.
Fluency of the performance	**5.45 (1.10)**	5.19 (1.04)	5.29 (1.07)	*p* = 0.032
Absorption	4.45 (1.32)	**4.99 (0.98)**	4.79 (1.15)	*p* < 0.001

Significant differences between professional and non-professional musicians were found in the mean values of both flow subscales. The fluency value of the professional group was significantly higher than in the non-professional group [*F*_(1, 361)_ = 4.65, *p* = 0.032, *d* = 0.23]. The mean value of the total sample in this scale was not significantly different to the value in the general population [*t*(362) = −1.03, *p* = 0.055]. However, while the professional musicians were not significantly different to the value of the general population at all [*t*(135) = 0.50, *n.s.*], the non-professional musicians showed a significantly lower mean value compared to the general population [*t*(226) = −2.92, *p* = 0.004, *d* = 0.39].

For the absorption scale, the non-professional group showed significantly higher mean values [*F*_(1, 361)_ = 19.40, *p* < 0.001, *d* = 0.48]. In comparison to the value in the general population, the mean scale value of the total sample was significantly higher [*t*(362) = 8.11, *p* < 0.001, *d* = 0.85]. However, the mean value of the professional musicians did not differ significantly from the value in the general population [*t*(136) = 1.37, *n.s.*], but the value of the non-professional musicians was significantly higher than in the general population [*t*(226) = 10.57, *p* < 0.001, *d* = 1.41].

Referring to the third research question, there were no significant differences in the Global Flow Score [*F*_(3, 327)_ = 2.52, *p* = 0.058] and the absorption scale [*F*_(3, 327)_ = 1.45, *n.s.*] between the instrumental groups (Table 3). In contrast, the fluency scale showed a significant difference across the instruments [*F*_(3, 327)_ = 3.07, *p* = 0.028, *d* = 0.38] with significantly higher values of the percussionists compared with the strings (*post hoc*, *p* = 0.022) and the brass (*post hoc*, *p* = 0.029).

**TABLE 3:** Mean values of the Flow Short-Scale (Rheinberg et al., 2003) (in brackets: standard deviation) by instrumental group (n.s., not significant; in bold: highest values).

	Global Flow Score mean (SD)	Fluency of the performance mean (SD)	Absorption mean (SD)
Strings (*n* = 215)	5.05 (0.98)	5.22 (1.10)	4.79 (1.17)
Woodwind (*n* = 58)	5.18 (0.73)	5.35 (0.96)	4.92 (0.99)
Brass (*n* = 43)	4.88 (0.89)	5.15 (1.04)	4.47 (1.21)
Percussion (*n* = 15)	5.58 (0.93)	**6.03 (1.06)**	4.92 (0.93)
Statistical differences between the instrumental groups	*p* = 0.058	*p* = 0.028	*n.s.*

### Performance-Specific Music Performance Anxiety and Self-Efficacy

To address the research questions about the MPA aspects, the results of the PQM are described first. The mean values of the PQM scales split by the two subgroups of professional and non-professional musicians are shown in Table 4. Significant differences across the two music groups have been found for PQM scales of the symptoms of MPA before the performance [*F*_(1, 348)_ = 12.25, *p* = 0.001, *d* = 0.39] and during the performance [*F*_(1, 348)_ = 11.18, *p* = 0.001, *d* = 0.37] with higher values for the non-professional musicians. The PQM scale functional coping after the performance was significantly higher for the non-professional than the professional musicians [*F*_(1, 348)_ = 8.43, *p* = 0.004, *d* = 0.27]. Also, the musical quality was rated significantly higher by the professional than by the non-professional musicians [*F*_(1, 348)_ = 14.80, *p* < 0.001, *d* = 0.57].

**TABLE 4:** Mean scale values of the performance-specific questionnaire on MPA (PQM) (mean with standard deviation) divided into professional and non-professional musicians; statistical differences between subgroups (n.s., not significant; in bold: highest values).

PQM scales		Professional musicians (*n* = 136) mean (SD)	Non-professional musicians (*n* = 227) mean (SD)	Total sample (*n* = 363) mean (SD)	Statistical difference between subgroups
Pre-performance	Functional coping	4.15 (0.71)	4.23 (0.66)	4.20 (0.68)	n.s.
	Symptoms of MPA	1.83 (0.88)	**2.18** (0.90)	2.05 (0.89)	*p* = 0.001
	Self-efficacy	3.69 (0.77)	3.75 (0.74)	3.74 (0.74)	n.s.
During performance	Functional coping	4.26 (0.71)	4.21 (0.61)	4.23 (0.65)	n.s.
	Symptoms of MPA	1.72 (0.82)	**2.01** (0.76)	1.90 (0.79)	*p* = 0.001
	Self-efficacy	4.01 (0.72)	4.02 (0.66)	4.02 (0.68)	n.s.
Post-performance	Functional coping	4.05 (0.76)	**4.28** (0.71)	4.19 (0.73)	*p* = 0.004
	Symptoms of MPA	1.76 (0.78)	1.71 (0.71)	1.72 (0.74)	n.s.
	Self-efficacy	3.92 (0.83)	4.05 (0.80)	4.05 (0.79)	n.s.
Self-rated musical quality		**4.66** (0.72)	4.34 (0.78)	4.33 (0.81)	*p* < 0.001
Personal importance of the performance		2.78 (0.74)	**3.07** (0.71)	2.96 (0.73)	*p* < 0.001
Compared difficulty		1.99 (1.00)	**2.41** (0.89)	2.26 (0.96)	*p* < 0.001
Perceived difficulty		2.80 (0.69)	**3.01** (0.75)	2.93 (0.73)	*p* = 0.007

Concerning the personal importance of the performance, the professional musicians had a significantly lower mean value than the non-professional musicians [*F*_(1, 358)_ = 12.89, *p* < 0.001, *d* = 0.40]. In comparison with other performances, the performance was rated as significantly more difficult by the non-professional musicians than by the professional musicians [*F*_(1, 354)_ = 16.53, *p* < 0.001, *d* = 0.62]. The mean value for the generally perceived difficulty of the performance was significantly lower for the professional musicians compared to the non-professional musicians [*F*_(1, 358)_ = 7.37, *p* = 0.007, *d* = 0.29]. The compared difficulty and the perceived difficulty correlated significantly with each other at *r* = 0.57 (Kendall Tau).

There were no significant differences between genders for any of the scales.

### Correlations Between the PQM and the Flow Scales

Considering the fourth research question, the correlation coefficients of the total sample (*n* = 363) with all scales of the PQM are shown in Table 5. The Global Flow Score correlated significantly with all PQM scales. Positive correlations were found with the PQM scales functional coping and self-efficacy between *r* = 0.35 and *r* = 0.63 and negative correlations with the PQM scale symptoms of MPA between *r* = −0.30 and *r* = −0.36).

**TABLE 5:** Correlations (Pearson’s r) between the PQM scales and the flow scales in the total sample (*n* = 363).

		Global Flow Score	Fluency of the performance	Absorption
PQM pre-performance	Functional coping	0.35**	0.39**	0.18**
	Symptoms of MPA	−0.30**	−0.42**	−0.01
	Self-efficacy	0.48**	0.42**	0.37**
PQM during performance	Functional coping	0.48**	0.55**	0.19**
	Symptoms of MPA	−0.32**	−0.46**	−0.01
	Self-efficacy	0.63**	0.64**	0.39**
PQM post-performance	Functional coping	0.56**	0.54**	0.39**
	Symptoms of MPA	−0.36**	−0.39**	−0.20**
	Self-efficacy	0.50**	0.47**	0.35**

Self-rated musical quality		0.38**	0.46**	0.12*

*p < 0.05;

**p < 0.01.

For the subscale of the fluency of the performance, there were similar positive correlations with the PQM scales functional coping and self-efficacy (between *r* = 0.39 and *r* = 0.64) and negative correlations with the PQM scale symptoms of MPA (between *r* = −0.39 and *r* = −0.46).

The absorption subscale had overall lower correlations with the PQM scales functional coping and self-efficacy between *r* = 0.18 and *r* = 0.39. For the PQM scales symptoms of MPA, there were no correlations between the absorption scale and the pre-performance scale and the scale during the performance, but there was a significant correlation of *r* = −0.20 with the post-performance scale.

The musical quality scale showed significant medium correlations of *r* = 0.38 with the Global Flow Score and for the subscales of *r* = 0.46 with the fluency scale, and a rather low correlation of *r* = 0.12 with the absorption scale.

The personal importance of the performance was significantly correlated with the Global Flow Score of *r* = 0.22 and with the absorption scale of *r* = 0.32 (both significantly at *p* < 0.01). No significant correlation was found for the fluency scale.

The compared difficulty to other performances showed no significant correlation with the Global Flow Score. The fluency scale and the absorption scale were negative correlated to a significantly low extent (both *r* = −0.17).

**FIGURE 1:**
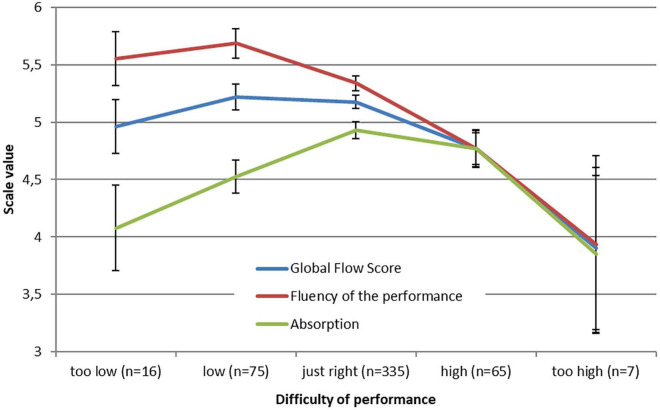
Mean values of the flow scales (with standard errors) at each rating of the difficulty ratings of the performance.

For the ratings of the general difficulty, the Global Flow Score was correlated to a significantly low extent with *r* = −0.13 and the fluency scale with *r* = −0.26. No correlation was found for the absorption scale. With a closer look at the flow scale distributions for this rating, the mean ratings at every scale answer showed that the scores did not simply follow a linear trend (Figure 1). At the difficulty ratings of “low” and “too low,” the mean scale values differ significantly from each other (*p* < 0.001), with the fluency scale showing the highest score and the absorption scale the lowest. When stating the difficulty as “high” or “too high,” the mean flow scale values did not differ from each other. At the rating of “just right,” the Global Flow Score and the absorption scale had their highest mean values. Both scales showed an inverted U-shape distribution.

### Predictors of Flow

To investigate possible influences of the particular variables on the experience of flow (research question five), linear regression analyses have been performed on the Global Flow Score. Factors included in the regression analysis were the three PQM scales at each time point before and during the performance as well as gender, age and the personal importance. To evaluate for possible shared variances between the PQM scales, the correlations between each scale at both times before and during the performance have been calculated (Table 6).

**TABLE 6:** Correlations (Pearson’s r) between the PQM scales before and during the performance.

		Before the performance			During the performance		
		Funct. coping	Sympt. of MPA	Self-efficacy	Funct. coping	Sympt. of MPA	Self-efficacy
Before the performance	Functional coping	−	−0.33**	0.42**	0.59**	−0.32**	0.41**
	Symptoms of MPA		−	−0.12*	−0.42**	0.76**	−0.28**
	Self-efficacy			−	0.36**	−0.15*	0.68**
During the performance	Functional coping				−	−0.52**	0.55**
	Symptoms of MPA					−	−0.31**
	Self-efficacy						−

*p < 0.05;

**p < 0.01.

The correlations between the PQM scales within the same time point showed significant low to moderate correlations. Because of the rather high correlations of the scales Symptoms of MPA and Self-Efficacy between before and during the performance, a single regression model may be influenced by the shared variances. Therefore, two regression analyses were performed separately for both time points.

The regression analysis before the performance identified possible associations between aspects prior to the performance and the occurrence of situational flow. For that, a hierarchical regression analysis were used. The first block contained the gender and the age as performance-independent individual demographic variables. In the second block, the personal importance of the performance were added. The third block included the variables of the first two blocks and the three PQM scales before the performance. The regression outcome is shown in Table 7. The first model was slightly significant and showed an explained variance of about 1% [*R*^2^ = 0.01, *F*_(2, 343)_ = 3.13, *p* = 0.045]. Age proved to be a significant predictor with a positive association, suggesting that older musicians appear to experience more flow. The second model increased significantly in the explained variance to 7% [*R*^2^ = 0.07, *F*_(3, 342)_ = 9.17, *p* < 0.001] indicating that the added variable of the individual importance is significantly associated with the flow experience. The third model showed an explained variance of 33% [adj. *R*^2^ = 0.33, *F*_(6, 339)_ = 29.09, *p* < 0.001] including significant predictors of the PQM scales self-efficacy and symptoms of MPA as well as the personal importance and the age. No influences of collinearities have been found (*VIF* between 1.02 and 1.15). The self-efficacy showed the highest prediction coefficient. With the exception of the symptoms of MPA, the parameters were positively associated with the flow. Although the PQM scale functional coping before the performance showed a significant correlation with the flow scale (see Table 5), this scale was not recognized as a significant factor in the regression model, indicating it to be a less important influence in the predictability of flow.

**TABLE 7:** Statistics of the linear hierarchical regression models on the Global Flow Score (*n* = 363) with variables prior to the performance.

Predictors of the General Flow Scale	*R*^2^ change	T	Std. beta coefficient	*p*-value
**Model 1 (*p* = 0.045)**	0.012*			
Gender		<1.0	0.05	n.s.
Age		2.11	0.12	0.036
**Model 2 (*p* < 0.001)**	0.057**			
Gender		<1.0	0.04	n.s.
Age		2.69	0.14	0.007
Importance of the performance		4.57	0.24	<0.001
**Model 3 (*p* < 0.001)**	0.265**			
Gender	<1.0	−0.03	n.s.	
Age		3.23	0.15	0.001
Importance of the performance		3.16	0.16	0.002
PQM functional coping (before)		1.66	0.08	n.s.
PQM symptoms of MPA (before)		−4.41	−0.22	<0.001
PQM self-efficacy (before)		7.85	0.41	<0.001
**Adjusted *R*^2^**	**0.33**			**<0.001**

*n.s., not significant; Significances of the R^2^ changes:*

*p < 0.05

**p < 0.001.

The second regression model has been performed on the Global Flow Scale with the three PQM scales during the performance along with the variables gender, age and personal importance of the performance. This analysis can identify potential changes of the influence of MPA during the performance on the experience of flow. The model showed an explained variance of 44% [adj. *R*^2^ = 0.44, *F*_(6, 343)_ = 46.92, *p* < 0.001] without any influences of collinearities (*VIF* between 1.05 and 1.80). The statistics of the analysis is shown in Table 8. Age and personal importance contributed to flow to a similar extent as in the regression before the performance. The PQM scales self-efficacy and functional coping during the performance were found as significant predictors. The self-efficacy during the performance showed the highest prediction coefficient. The scale Symptoms of MPA during the performance was not found as a significant predictor, even if it was significantly correlated with the flow experience.

**TABLE 8:** Statistics of the linear regression analysis on the Global Flow Score with the PQM scales during the performance (*n* = 363).

Predictors of the General Flow Scale	T	Std. beta coefficient	*p*-value
Gender	<1.0	−0.04	n.s.
Age	2.63	0.11	0.009
Importance of the performance	3.35	0.15	<0.001
PQM functional coping (during)	3.28	0.18	0.001
PQM symptoms of MPA (during)	−1.91	−0.09	n.s.
PQM self-efficacy (during)	9.11	0.47	<0.001

*n.s., not significant; Adjusted R^2^ = 0.44, p < 0.001.*

## Discussion

The aim of the present study was to examine the flow experience during a performance, as well as the symptoms of MPA, coping with MPA and self-efficacy before, during and after the same performance, and to identify mutual influences.

### Flow

In the present study, a questionnaire was used with the flow short scale, which relates to the specific experience during a performance. The Global Flow Scale of the entire sample of professional and non-professional orchestral musicians was significantly higher than the value of the general population. However, in more detail, there was only a significant difference in that flow scale for the non-professional musicians compared to the mean value in the general population with higher flow experience in the non-professional musicians. The professional musicians showed a similar extend of flow as the general population.

In our study, the absorption scale showed a higher value for the non-professional orchestral musicians than for the professional musicians. Absorption means that there is a high degree of correspondence with the activity of making music, i.e., the non-professionals were more absorbed in making music themselves and had a different sense of time. The professional orchestral musicians on the other hand rated higher on the fluency scale. Fluency expresses the flowing and automated character of playing and it makes sense that this is more present among professional musicians given their higher level of expertise. This confirms the findings of Kutepova-Bredun (2018) who also found higher values for fluency among elite musicians than in amateur musicians, while other authors found that flow was not experienced more often among elite musicians compared to amateur musicians (Sinnamon et al., 2012).

In conclusion, it can be assumed that the professional musicians may have experienced a higher fluency than the non-professional musicians due to their greater experience, but the non-professional musicians can immerse themselves better in the music. It may not seem so surprising that non-professional musicians be more able to immerse themselves in the music than professional musicians do. Especially when people do not live from making music, they are often particularly emotionally involved and joy is the main focus. This could be a reason why they are more likely to be completely absorbed in the music. However, the flow experiences of the professional musicians did not differ from the experiences of the general population in other activities at all. Where Cohen and Bodner (2021) found high frequent flow occurrences in professional musicians, the amount of flow experience itself was not different to the general population. The research question on the extent of the flow experience must therefore be answered differently for professional and non-professional musicians.

The finding of Habe et al. (2019, 2021) that flow was experienced more in male than in female musicians was not confirmed in this study. The results revealed no differences between genders in terms of flow in live performances.

Regarding the research question on flow in different instrumentalists, the results showed that there were no differences in global flow experiences between the investigated instrument groups, confirming the findings of Wrigley and Emmerson (2013) in examination performances. Only in the fluency scale, significantly higher values were found for the percussionists compared with the strings and the brass. This difference seems plausible, especially since the playing processes, especially in the percussion, are highly automated and make the experience of fluency while playing particularly likely. Rhythmic patterns also often have a high repeatability, so that a trance-like fluency can be achieved by playing continuous rhythms and a balance between skill and challenge can be obtained more easily.

The perceived difficulty of the performance showed an inverted U-shape relationship with the flow experience confirming the findings of Rheinberg et al. (2003). This specific relation was especially the case for the absorption scale. For a higher sense of immersion, a right balance of the demands seems to be necessary. In contrast, the fluency scale had the lowest U-shaped relationship, with scores higher the lower the performance difficulty was rated. In order to perceive fluency, the demands do not have to be too difficult. This correlation could also support the findings that percussionists had the highest scores for fluency.

### Music Performance Anxiety

The PQM scales showed that the non-professional musicians had higher symptoms of MPA before and during the performance but not after the performance. This indicated that the non-professional musicians seemed to be more affected by their MPA than the professional musicians were. This may be due to the higher degree of experience of the professional musicians since the non-professional musicians may perform less frequently. Non-professional musicians are also less musically educated than professional musicians are and may be more unsure whether they will sing and play well during the performance. This uncertainty contributes to the fact that MPA is increased. On the other hand, non-professional musicians are more satisfied with their performance and show better functional coping post-performance than professional musicians do. If you focus on the professional musicians, our results suggest that professional musicians need help with developing skills for post-performance analysis.

After the performance, the non-professional musicians were at the same level as the professional musicians in the symptoms of MPA scale, but also higher in the functional coping scale. The non-professional musicians seemed to cope successfully with the experienced MPA before, during and after the performance.

### Music Performance Anxiety and Flow

It was expected that the MPA during the performance might show the known negative relation with the flow experience. This was the case in the scale “symptoms of MPA” in the PQM questionnaire before, during and after the performance, indicating the significantly negative correlations at all three times and confirming the hypothesis.

This result has been found in the literature in several studies before. The significant correlations confirmed the findings of Kirchner et al. (2008) that less MPA is associated with more flow experiences.

The results of the regression analysis with the parameters before the performance give clues as to which MPA aspects can predict the occurrence of flow. The regression model showed a high proportion of explained variance. It confirms the negative influence of the symptoms of MPA on the flow. The age was also found as a significant predictor with older musicians having stronger flow experiences. Particularly in the field of orchestral music, this could be related to the fact that fluency and the automation of playing increases with increased experience in orchestral service. This indicates that greater musical experience seems to have a positive influence on the occurrence of flow.

Another factor was the individual importance of the performance. The more important the performance was for the musicians, the stronger the flow experience. It may be that a certain level of importance to the musicians can prevent boredom from developing, which in turn may reduce the experience of flow. This is supported by the findings of Antonini Philippe et al. (2021) that motivation to perform is a relevant factor in promoting flow.

The most essential influencing factor in the regression model before the performance appears to be the self-efficacy. The important role of self-efficacy in connection with MPA has been extensively studied (Wrigley and Emmerson, 2013; Miksza and Tan, 2015). The findings show that the self-efficacy regarding a particular performance can also increase the occurrence of flow during the performance. A certain level of confidence may lead the player to approach the performance as a challenge. This may prevent too much concentration on negative aspects of the performance and increase the chance of immersing oneself in the performance. In addition, since confidence in one’s abilities is an essential component of self-efficacy, the findings of Antonini Philippe et al. (2021) confirm our findings that higher self-efficacy can promote flow.

Before the performance, despite the fact that there is a significant correlation of the PQM scale functional coping with the flow, it has not been found as a significant predictor in the regression analysis of the third model. This scale represents a certain kind of coping with the appearance of individual MPA prior to the performance and seems to have less influence on the occurrence of flow during the performance even if the correlation was slightly higher than for the PQM scale symptoms of MPA.

During the performance, the regression analysis provides the relationship of how MPA can situationally affect the occurrence of flow. The analysis showed that the PQM scale symptoms of MPA was not found as a significant predictor. Instead, the scale functional coping appeared to be of significant influence. Where the symptoms of MPA prior to the performance had a significant effect on the experience of flow, during the performance they seemed to be pushed into the background. Thus, the positive coping with MPA was of more importance during playing. Additionally, the self-efficacy was again found as the highest predictor of flow. Even during the performance, the results confirm that confidence in one’s own abilities can increase the occurrence of flow.

After the performance, the correlations between the PQM scales and the flow scale showed certain connections. Unlike the other time points, the occurrence of flow may affect the experience of MPA. Therefore, the feeling of flow during the performance seems to increase the positive coping and handling of MPA and decrease the symptoms of MPA. It also seems to increase the self-efficacy. After all, the experience of flow during the performance was positively correlated with the ratings of the musical quality. These findings clearly indicate a very positive effect of having flow experiences during a performance on the perceived performance outcome. A positively experienced performance, however, can then increase self-confidence for future performances and strengthen the individual’s ability to cope with MPA.

A limitation of this study might be that it would have been of interest to collect more detailed information from the musicians and the orchestras in order to perform a more sophisticated analysis. However, in this study, the focus was on the general relationship between flow and MPA in standard live performances. Further studies could additionally investigate the changes over multiple performances.

## Conclusion

In this study, it has been confirmed that flow represents an interesting focus for coping with MPA. Especially during musical education, the focus should be more directed to enabling experiences of flow than to avoiding MPA. At the same time, this promotes a positive and healthy self-awareness when making music and shifts MPA onto a performance-enhancing level.

Since MPA before and during a performance seems to be a significant predictor of flow, coping strategies for MPA should be taught to music students during their studies. Thus, it could be an important goal of performance preparation to achieve flow and the facilitating form of MPA at the same time. Music students should be supported in building a positive self-efficacy in order to enable flow. An important approach is to strengthen self-efficacy before a performance, especially since this not only promotes flow during the concert, but also results in positive self-awareness after the concert. This can build strong self-efficacy for the next performance and promotes an overall positive self-concept with regard to making music in performance situations. In summary, the results provide interesting starting points for implementations in practice.

## Data Availability Statement

The raw data supporting the conclusions of this article will be made available by the authors, without undue reservation.

## Ethics Statement

The studies involving human participants were reviewed and approved by the Ethics Committee of the University Medical Center Freiburg. Written informed consent for participation was not required for this study in accordance with the national legislation and the institutional requirements.

## Author Contributions

FK mainly carried out the data collection. CS and MN performed the statistical analyses. All authors co-wrote the manuscript, contributed extensively to the work presented in this manuscript, and contributed to the conception and design of the study.

## Conflict of Interest

The authors declare that the research was conducted in the absence of any commercial or financial relationships that could be construed as a potential conflict of interest.

## Publisher’s Note

All claims expressed in this article are solely those of the authors and do not necessarily represent those of their affiliated organizations, or those of the publisher, the editors and the reviewers. Any product that may be evaluated in this article, or claim that may be made by its manufacturer, is not guaranteed or endorsed by the publisher.
